# Mirabegron induces selective changes in the faecal microbiota of HFHFr rats without altering bile acid composition

**DOI:** 10.3389/fphar.2025.1547749

**Published:** 2025-04-14

**Authors:** Roger Bentanachs, Lluïsa Miró, Patricia Ramírez-Carrasco, Rosa M. Sánchez, Manuel Bernabeu, Concepció Amat, Marta Alegret, Anna Pérez-Bosque, Núria Roglans, Juan C. Laguna

**Affiliations:** ^1^ Department of Pharmacology, Toxicology and Therapeutic Chemistry, School of Pharmacy and Food Science, University of Barcelona, Barcelona, Spain; ^2^ Institute of Biomedicine IBUB, University of Barcelona, Barcelona, Spain; ^3^ Department of Biochemistry and Physiology, School of Pharmacy and Food Science, University of Barcelona, Barcelona, Spain; ^4^ Institute for Nutrition and Food Safety Research INSA-UB, University of Barcelona, Barcelona, Spain; ^5^ Spanish Biomedical Research Centre in Physiopathology of Obesity and Nutrition (CIBEROBN), Instituto de Salud Carlos III (ISCIII), Madrid, Spain; ^6^ Department of Biotechnology, Institute of Agrochemistry and Food Technology, National Research Council (IATA-CSIC), Paterna, Spain

**Keywords:** MASLD, SLD, β3 agonists, UCP1, akkermansia, *Clostridium*

## Abstract

**Introduction:**

Metabolic dysfunction-associated steatotic liver (MASL), the initial, asymptomatic stage of the metabolic dysfunction-associated steatotic liver disease, is directly involved in the progression to steatohepatitis. Healthy lifestyle and dietary measures are currently the only treatments for MASL. Given the high prevalence of MASL in the human population, candidate drugs for its prevention or treatment should have an acceptable safety profile. Repurposing drugs already in clinical use could help to identify effective and safe drug treatments for MASL. We have characterized a high-fat, high-fructose rat dietary model of simple hepatic steatosis to evaluate the potential anti-steatotic effect of mirabegron, which is already in clinical use for the treatment of overactive bladder. We have previously reported that mirabegron administration was unable to reduce liver triglyceride content in our rat model.

**Methods:**

In the present work, we analyse stored liver, adipose tissue (perigonadal and brown), serum and faecal samples from our previous study and present new biochemical, faecal metabolomic and microbiome data.

**Results and discussion:**

We show that oral administration of mirabegron significantly increases the expression of uncoupling protein 1 in brown adipose tissue and β3-Adrenergic receptor protein in perigonadal white adipose and liver tissues. Furthermore, mirabegron treatment changes the relative abundance of several genus and families of rat faecal microbiota, albeit without restoring the global biodiversity and evenness indexes observed in control rats, as well as faecal bile acids composition. These changes are probably due to a direct effect of mirabegron on the gut microbiome, rather than being mediated by changes in bile acid induced by drug treatment.

## 1 Introduction

Mirabegron (MBG) is a selective β3 adrenergic receptor (β3-RA) agonist in clinical use for the treatment of overactive bladder (Yuanzhuo et al., 2022). In healthy humans, it has been described that chronic mirabegron administration increases brown adipose tissue (BAT) metabolic activity and adiponectin blood concentration (O’Mara et al., 2020), enhancing resting energy expenditure and lipid oxidation (Nahon et al., 2020; Ma et al., 2024). There has been some concern about the possibility that mirabregon administration could potentiate the development of atherosclerosis and associated cardiovascular diseases, generated from the analysis of preclinical data obtained from murine models (mice genetically deficient in apolipoprotein E or LDL receptor) (Sui et al., 2019). However, a recent study by Zhixiong Ying et al., which used a humanized lipoprotein mouse model (APOE*3-Leiden.CETP), with lipoprotein metabolism similar to humans, demonstrated that treatment with mirabegron does not promote the development of atherosclerosis in this model (Ying et al., 2023). Thus, mirabegron could potentially be used in a clinical setting to improve the control of metabolic diseases associated to excess tissue lipid deposition in humans (Ma et al., 2024).

Metabolic dysfunction-associated steatotic liver (MASL) is diagnosed by the accretion of neutral lipids (triglycerides and cholesteryl-esters) in more than 5% of hepatic parenchymal cells. It is the initial, asymptomatic stage of metabolic dysfunction-associated steatotic liver disease (MASLD), which is the most prevalent liver disease, affecting more than 30% of the population worldwide (Powell et al., 2021; Targher et al., 2021; Riazi et al., 2022; Rinella et al., 2023). Resmetirom, a thyroid hormone receptor beta-selective agonist, can improve liver fibrosis and has recently received United States Food and Drug Administration approval for the treatment of the advanced form of MASLD, metabolic dysfunction-associated steatohepatitis (MASH) (Harrison et al., 2024). However, there is currently no drug approved for the treatment of isolated steatosis, with options limited to the implementation of a healthy lifestyle, including an increase in physical activity and the adoption of dietary measures, such as the reduction of simple sugars consumption and adherence to healthy dietary patterns, such as the Mediterranean diet (El-Agroudy et al., 2019). Unfortunately, these changes in lifestyle habits are difficult to sustain in the long run. As liver triglyceride accretion is directly involved in the progression of MASL to MASH (Semova and Biddinger, 2021) and given the high prevalence of MASL in the human population, candidate drugs for its prevention/treatment should present an acceptable safety profile. Repurposing drugs already in clinical use with a good safety record reduces the development costs with substantial savings in the preclinical and clinical Phase I and II steps, also shortening the time invested in the approval of a new therapeutic indication (Pushpakom et al., 2018), representing a good option in the pathway of finding an effective and safe drug-treatment for MASL.

Dysregulation of the gut microbiota is a hallmark of MASLD (Quesada-Vázquez et al., 2020; Vallianou et al., 2022), and it has been linked to increased disease severity through dysbiosis and loss of key metabolic functions provided by commensal bacteria (Aron-Wisnewsky et al., 2020). Although research on the effects of β3-AR stimulation on the microbiota remains very limited, β3-AR agonist treatment has been shown to alter gut microbiota composition in several models of obesity and thermogenesis (Paz et al., 2022; Samanta et al., 2024).

In the present work, we have used our previously characterized high fat-high fructose (HFHFr) rat isolated steatosis dietary model (Velázquez et al., 2022a), to evaluate the potential anti-esteatotic effect of several drugs already in clinical use, such as bempedoic acid, pemafibrate and mirabegron (Velázquez et al., 2022b; Bentanachs et al., 2024). As we reported previously (Bentanachs et al., 2024), mirabegron administration failed to reduce liver triglyceride content in our rat model. However, we present experimental data showing that mirabegron administration was associated with significant changes in the adipose tissue and the rat faecal microbiome.

## 2 Materials and methods

### 2.1 Animals and experimental design

Two-month-old female Sprague Dawley rats (Envigo, Barcelona, Spain) weighing 147 ± 5 g were housed two per cage under the following conditions: 40%–60% humidity, 20◦C–24◦C temperature, and 12 h light/dark cycles. Forty rats were randomly assigned to five groups (8 per group) and fed the following diets for 3 months: (i) the control group (CT) was fed a regular chow diet (2018 Teklad Global rodent diet, Envigo, Barcelona, Spain), with free access to water; (ii) the HFHFr group was fed a high-fat diet (HFD) (Teklad Custom Diet TD. 180456, Envigo, Madison, WI, United States), with free access to a 10% w/v fructose solution; (iii) the MBG group (10 mg/kg/day) was fed the HFHFr diet and treated during the third month. As described previously (Bentanachs et al., 2024), drug-treated rats received the HFD solid food with the drugs incorporated into it, at a concentration to provide the aforementioned daily dose. Solid food and liquid consumption were controlled three times a week, and body weight was recorded once a week. After adjusting for the actual HFD consumption during the last month of drug administration, the mirabegron dose received by the rats, expressed as mg of drug/kg of rat weight/day, was 10.2. Mirabegron was provided by Interquim SA, Spain. All procedures involving animals were carried out according to institutional guidelines established by the Bioethics Committee of the University of Barcelona (Autonomous Government of Catalonia Act Biomedicines 2022, 10, 1517 3 of 19 5/21 July 1995). The Animal Experimentation Ethics Committee, University of Barcelona, approved all experiments performed on animals (approval no. 10106).

### 2.2 Sample collection

At the end of the treatment, rats fasted for 2 h were anesthetized with ketamine/xylazine (9 mg/40 μg per 100 g of body weight, respectively), blood was collected into micro-tubes (Sarstedt AG and Co., Nümbrecht, Germany) through cardiac puncture and the rats were euthanized by exsanguination. Samples of liver, BAT, subcutaneous and perigonadal white adipose tissue (sWAT and pWAT, respectively), colon tissue, and faecal content were collected, immediately frozen in liquid nitrogen and stored at −80°C until needed for biomolecular assays. Serum samples were obtained through cardiac puncture and centrifuged at 10,000 × g for 5 min at room temperature into micro-tubes (Sarstedt AG and Co., Nümbrecht, Germany).

### 2.3 Serum analytes

Serum non-esterified free fatty acids (NEFA) were determined using a colorimetric kit from BiooScientific (Austin, TX, United States). The glycerol determination kit was from SpinReact (Girona, Spain). All other serum analytes were determined as described in Bentanachs et al. (2024).

### 2.4 Fatty acid β-oxidation activity

Total fatty acid β-oxidation activity was determined as described by Lazarow (1981), using 30 µg of postnuclear supernatant from the liver samples.

### 2.5 Faecal bile acids analysis

The concentrations of 10 bile acids (BA) (cholic acid, chenodeoxycholic acid, deoxycholic acid, lithocholic acid, ursodeoxycholic acid, α-muricholic acid, β-muricholic acid, hyocholic acid, hyodeoxycholic acid, and murideoxycholic acid) in rat faeces were determined by ultra-high-performance liquid chromatography coupled to tandem mass spectrometry (UHPLC-MS/MS). Briefly, 50 mg of lyophilized sample was mixed with 50 µL of internal standard (containing isotopically labeled bile acids) and 500 µL of isopropanol. After vortexing for 1 min, and centrifuging at 13,500 rpm for 5 min, the supernatant was diluted 10-fold with a mixture of water and methanol at a 1:2 ratio. Finally, 10 µL of the diluted extract was injected in the UHPLC-MS/MS system.

The instrumental analysis was performed on an Acquity I-Class UPLC liquid chromatograph (Waters, MA, United States) coupled to a Xevo TQ-S micro mass spectrometer (Waters, MA, United States), equipped with a Z-spray electrospray source. Nitrogen 99.99% was used as drying and nebulizing gas, and argon 5.0 as collision gas. Source was kept at 150°C and the desolvation gas was set at a flowrate of 1200 L/h and a temperature of 450°C, whereas the cone gas was set at 50 L/h. Positive and negative ionization mode was used with a capillary voltage of 3 kV in both cases. Masslynx software V4.1 was used for data acquisition and processing.

Separation was carried out on an Acquity BEH C18 column (Waters, 2.1 × 100 mm, 1.7 µm) with a mobile phase composed of a mixture of methanol and water, both with 1 mM ammonium formate and 0.01% formic acid. The column temperature was set at 30°C and the flow rate was set at 0.2 mL/min. The chromatographic gradient was as follows (aqueous percentage is indicated): 0 min, 35%; 0.5 min, 35%; 10.5 min, 26%; 12.5 min, 10%; 12.51 min, 0%; 14 min, 0%, 14.01, 35%; 16 min, 35%. Calibration standards containing a mixture of the corresponding analytes were prepared at 10 concentration levels and processed together with the samples. Quantification was performed by external calibration curve analysis, using the ratio between the peak areas of the analyte and the corresponding internal standard as the response. Calibration curves were constructed by least squares linear regression of the response weighted by the inverse of the concentration. The hydrophobicity index (HI) was calculated as described by Heuman et al. (1989).

### 2.6 Western blot analysis

Tissue samples for Western blot analysis were homogenised with a Polytron PT 1200E homogeniser in lysis buffer containing proteases, phosphatases, and deacetylase inhibitors, and incubated for 1.5 h at 4°C. Samples were then centrifuged at 15,000 x g for 15 min at 4°C, and the supernatants were collected. Protein concentrations were determined by the Bradford method (Bradford, 1976).

Western blots were carried out using 20 µg of total protein for pWAT and BAT samples, or 80 µg for liver samples, were subjected to SDS-polyacrylamide gel electrophoresis. Proteins were then transferred onto Immobilon polyvinylidene difluoride transfer membranes (Millipore, Billerica, MA, United States), and blocked for 1 h at room temperature with blocking solution (SP 7000; WestVision, CA, United States). Membranes were then incubated with specific primary antibodies (UCP1 sc-518024, 1:500; β3ADR sc-515763, 1:200; and ATGL sc-365278, 1:1000 from Santa Cruz Biotech; HSL #4107, 1:1000 and, p-HSL #4126, 1:1000, from Cell Signaling). Detection was performed using the Immobilion Western HRP substrate Peroxide Solution^®^ (Millipore, Billerica, MA, United States). To confirm the uniformity of protein loading, blots were incubated with anti-vinculin (Santa Cruz Biotech. Dallas, TX, United States) antibody as a control for total protein extracts.

### 2.7 RT-qPCR analysis

Total RNA was extracted from liver and colon (50–100 mg of each sample) using a phenol-based method according to the manufacturer’s instructions (Trisure^®^ reagent, Bioline, Meridian Biosciences, Cincinnati, OH, United States). RNA concentration was determined by measuring absorbance at 260 nm, while the 260/280 nm absorbance ratio was used to analyse RNA quality. For real-time polymerase chain reaction (RT-qPCR), 1 μg RNA was reverse transcribed into cDNA using the Moloney Murine Leukemia Virus Reverse Transcriptase (MLV-RT; Invitrogen, Carlsbad, CA, United States), random hexamers (Roche, Meylan, France) and dNTP (Sigma-Aldrich, St. Louis, MO, United States). The specific mRNAs were assessed in the StepOnePlus Real-Time PCR System Thermal Cycling Block (Applied Biosystems, Foster City, CA), using 100 µM of each specific primer, 10–20 ng of cDNA, and SYBR Green Perfect Master Mix with ROX in 96 well-plates. mRNA expression was calculated using the recommended 2^−ΔΔCt^ method. The *β-actin* was used as the housekeeping gene to normalize the results. The primer sequences, Genbank TM number and PCR product lengths are listed in (Bentanachs et al., 2024) and, for *muc2* and *tnfα*, in (Miró et al., 2020).

### 2.8 Faecal microbiota analysis

Microbiota DNA was extracted and processed as described by Rosell-Cardona et al. (Rosell-Cardona et al., 2022). Microbiome bioinformatics was performed using QIIME2 2023.7 (Bolyen et al., 2019). The raw sequence data were demultiplexed and quality filtered using the q2-demux plugin, followed by denoising with DADA2 (Callahan et al., 2016). All amplicon sequence variants (ASVs) were aligned with MAFFT (Katoh et al., 2002) and used to construct a phylogeny with FastTree2 (Price et al., 2010). Alpha-diversity metrics (Shannon index and Faith’s Phylogenetic Diversity), and beta-diversity metrics such as Bray-Curtis dissimilarity were estimated using q2-diversity plugin after samples were rarefied (Roger Bray and Curtis, 1954; Faith, 1992). Taxonomy assignment to ASVs was performed using the q2-feature-classifier (Bokulich et al., 2018) classify-sklearn naive Bayes taxonomy classifier against the Greengenes2 reference sequences (McDonald et al., 2024). Only taxa with a percentage of reads higher than 0.001% were included in the Microbiota analysis. Permutation Multifactorial Analysis of Variance (PERMANOVA) was performed using Bray-Curtis dissimilarity matrix in R with the “adonis” function. Principal Coordinate Analysis (PCoA) ordination based on Bray-Curtis dissimilarity was used to visualize the dispersion of microbial community among groups. Linear discriminant analysis effect size (LEfSe) method with logarithmic discriminant analysis (LDA) scores was used to identify taxa differing in relative abundance among groups (Segata et al., 2011). For LEfSe analysis, the Kruskal–Wallis test, an all-against-all strategy was applied with a *P* < 0.05 and a LDA score threshold of 4.0. Both analyses were performed in RStudio (2023.09.1 Build 494^©^ 2009–2023 Posit Software, PBC) using the vegan and phyloseq-class packages (Cao et al., 2022).

### 2.9 Quantification of faecal short-chain fatty acids

The concentration of short-chain fatty acids (SCFA) was determined using an Agilent GC 7890B-5977 gas chromatography-mass spectrometry (GC–MS) system, equipped with a multipurpose sampler (Gerstel MPS). The GC column employed was an Agilent DB-FATWAX with dimensions of 30 m × 0.25 mm × 0.25 μm, operating in split mode. Helium served as the carrier gas with a flow rate of 1 mL per minute. Samples of each experimental group were analysed separately. Standard calibration curves were previously obtained for all SCFAs analyzed.

GC-MS analysis has been made following the procedure described by Eberhart et al. (2021). 3-methylvaleric acid was employed as an internal standard at a concentration of 5 mM. Standard curves for acetic, propionic and butyric acid were utilized for quantifying SCFA in the samples. For sample preparation, 200 mg of faeces from each rat were weighed, resuspended in 1 mL of PBS and centrifuged at 13,000 rpm for 5 min. Subsequently, 200 μL of supernatant samples were combined with 800 μL of the internal standard solution, 1 mL of diethyl ether, and a spoonful of Na_2_SO_4_. The mixture was vortexed for 10 s and centrifuged at 1,500 × g for 2 min at 4°C. Finally, 200 μL of the upper phase was transferred to a chromatography vial for analysis.

### 2.10 Statistical analysis

Results are expressed as mean ± standard deviation (SD). Significance was established by one-way ANOVA and Sídák’s multiple comparisons test applied as a post-hoc test. (GraphPad Software version 10, San Diego, CA, United States). When the SD of the groups was different according to the Brown–Forsythe test, the data were log-transformed and ANOVA was rerun, or the corresponding non-parametric test (Kruskal–Wallis) was applied. For microbiota analysis, Grubb’s test was applied to identify and eliminate outliers. Additionally, Levene’s test was used to assess the homogeneity of variance and the Shapiro–Wilk test to verify data normality across groups. Post hoc analyses were performed using Fisher’s Least Significant Difference (LSD) test. The correlation between faecal microbiota and SCFA was performed using the Spearman correlation, and network plot was generated using Cytoscape v 3.10.1. Statistical significance was considered when P *<* 0.05.

## 3 Results

### 3.1 MBG elicited specific changes in the liver and in pWAT expression of several genes related to lipid catabolism

**FIGURE 1:**
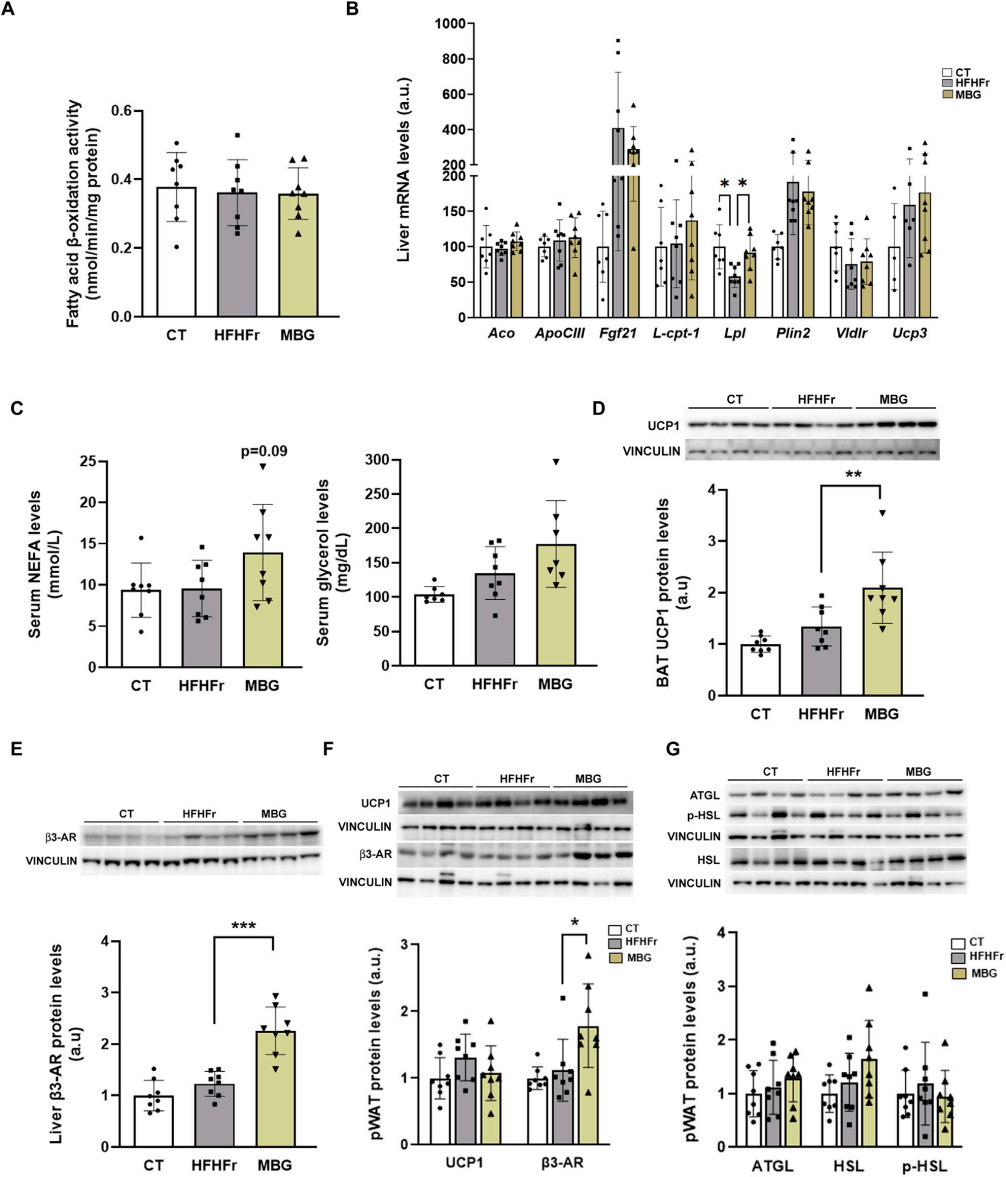
Mirabegron increased the UCP1 protein content in BAT tissue and the amount of β3-AR protein in liver and pWAT. **(A)** Fatty acid β-oxidation activity in liver samples from the CT, HFHFr and MBG experimental groups. **(B)** Relative liver mRNA levels of *aco, apo CIII*, *fgf21*, *l-cpt-1*, *lpl*, *plin2*, *vldlr* and *ucp3* genes in samples from the CT, HFHFr and MBG experimental groups. **(C)** Serum concentrations of NEFA and glycerol in samples from the CT, HFHFr and MBG experimental groups. **(D)** Relative content of UCP1 protein in BAT samples from the CT, HFHFr and MBG experimental groups. On the upper side of the figure, representative Western blot bands corresponding to the three different study groups are shown. **(E)** Relative content of β3-AR protein in liver samples from the CT, HFHFr and MBG experimental groups. On the upper side of the figure, representative Western blot bands corresponding to the three different study groups are shown. **(F)** Relative content of UCP1 and β3-AR proteins in pWAT samples from the CT, HFHFr and MBG experimental groups. On the upper side of the figure, representative Western blot bands corresponding to the three different study groups are shown. **(G)** Relative content of AGTL, phosphor- and total-HSL proteins in pWAT samples from the CT, HFHFr and MBG experimental groups. On the upper side of the figure, representative Western blot bands corresponding to the three different study groups are shown. Quantitative results are presented as bar plots with individual values, showing the mean ± SD of 7-8 animals/group. *P < 0.05, **P < 0.01, ***P < 0.001.

We have previously shown that MBG at a daily dose of 10 mg/kg for 1 month did not modify liver or serum triglyceride concentrations in a rat model of isolated steatosis induced by a high-fat diet supplemented with liquid fructose (Bentanachs et al., 2024). In contrast, it has been shown that BRL37344, another β3 receptor agonist, exerts liver-specific effects on a different experimental MASLD model, effectively reducing liver triglyceride accumulation in rats fed a high-fat diet. The proposed mechanism of action of BRL37344 involved the increase in peroxisome proliferator activated receptor α (PPARα) expression and activity (Wang et al., 2020). As shown in Figures 1A, B, in our model mirabegron administration changed neither fatty acid β-oxidation activity, a catabolic pathway enhanced by PPARα activation, nor the expression of liver-carnitine palmitoyl transferase I (*l-cpt-1)* and acyl-CoA oxidase (*aco*), genes coding for enzyme proteins that directly control mitochondrial and peroxisomal fatty acid β-oxidation. Moreover, the liver expression of most genes under the transcriptional control of PPARα, such as apolipoprotein CIII (*apo CIII*), fibroblast growth factor 21 (*fgf21*), perilipin 2 (*plin2*), VLDL receptor (*vldlr*) and uncoupling protein 3 (*ucp3*) remained unchanged. In contrast, the relative concentration of lipoprotein lipase (*lpl*) mRNA was significantly increased by mirabegron treatment (x1.6-fold vs. HFHFr) (Figure 1B).

MBG non-significantly increased serum NEFA (x1.64) and glycerol (x1.31) concentrations, pointing to an activation of WAT lipolysis (Figure 1C). Moreover, similarly to what has been described in humans (Baskin et al., 2018; O’Mara et al., 2020), mirabegron increased the UCP1 protein content in BAT, not in pWAT. Mirabegron also increased the relative amount of β3-AR protein in pWAT and liver (Figures 1D–F), similarly to what has been described for BRL37344 (Wang et al., 2020), although this effect did not translate in an increase in the expression of adipose triglyceride lipase (AGTL), and in the phosphorylation of hormone sensitive lipase (HSL) (Figure 1G). Overall, although MBG was unable to reverse liver lipid deposition in our HFHFr rat model at a 10 mg/kg/day p. o. dose, its administration to HFHFr rats elicited measurable biological changes in liver, BAT and pWAT rat tissues.

### 3.2 MBG treatment did not lead to significant changes in faecal BA composition of HFHFr rats

**FIGURE 2:**
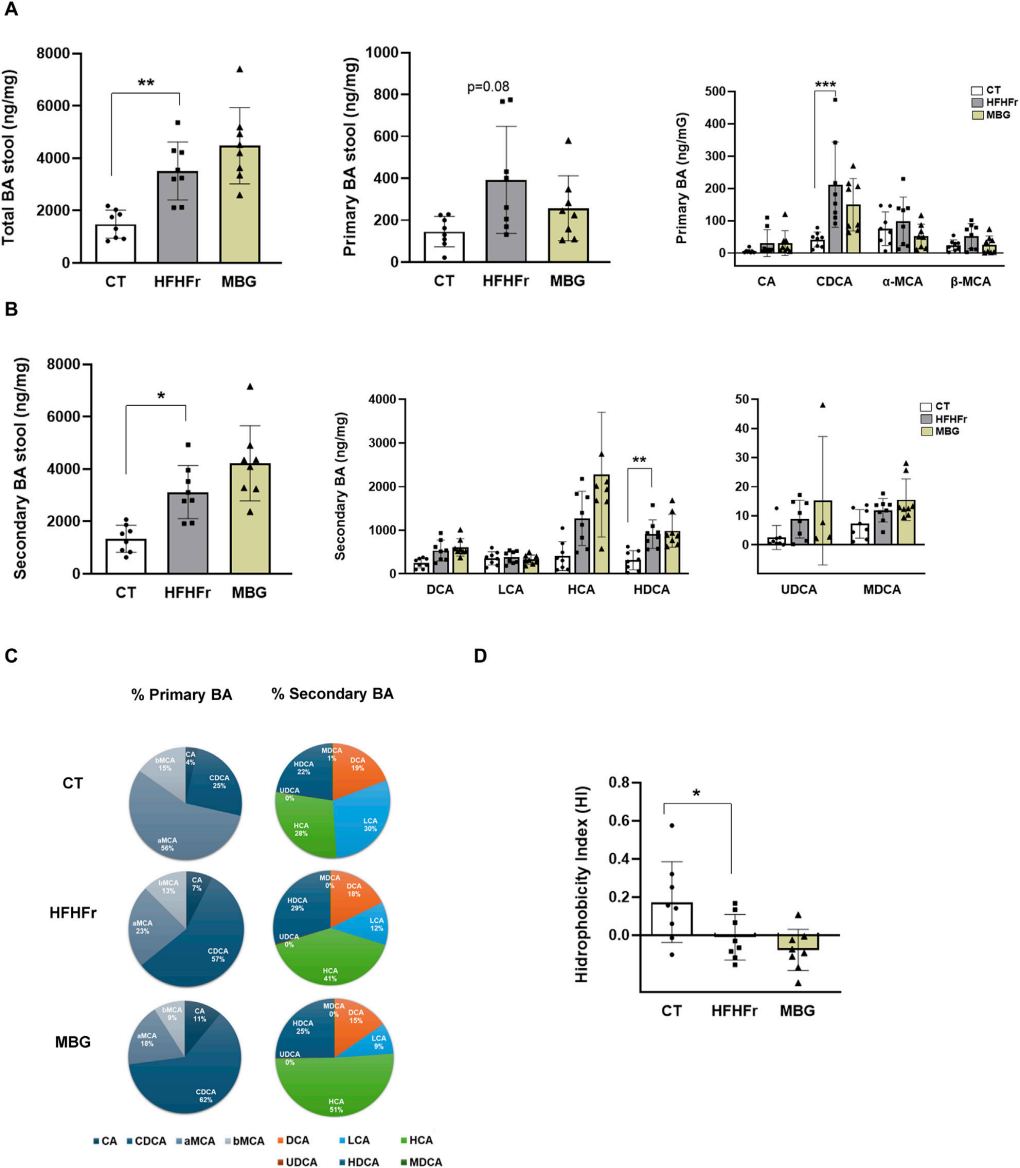
MBG administration did not change the fecal concentration of primary and secondary bile acids (BA) in HFHFr rats. **(A)** Concentrations of total BA, total primary-BA and individual primary-BA (CA, CDCA, α-MCA and β-MCA) in stool samples from the CT, HFHFr and MBG experimental groups. **(B)** Concentrations of total secondary-BA and individual secondary-BA (DCA, LCA, HCA, HDCA, UDCA and MDCA) in stool samples from the CT, HFHFr and MBG experimental groups. **(C)** Cyclograms representing the relative proportion of individual BA in stool samples from the CT, HFHFr and MBG experimental groups. **(D)** Hydrophobicity Index (HI) of total BA in stool samples from the CT, HFHFr and MBG experimental groups. Quantitative results are presented as bar plots with individual values, showing the mean ± SD of 7-8 animals/group. *P < 0.05, **P < 0.01, ***P < 0.001.

Based on reports that MBG induces changes in BA metabolism in humans, reducing plasma concentrations of glycochenodeoxycholate, glycocholic acid, glycodeoxycholate, and taurodeoxycholic acids (Baskin et al., 2018), we determined the BA composition of faecal samples from HFHFr rats treated with MBG. As shown in Figure 2, MBG treatment did not significantly modify the absolute and relative concentrations of total, primary and secondary BA present in the faecal samples analysed, as well as the value of the global hydrophobicity index.

### 3.3 MBG induced selective changes in the faecal microbiota composition of HFHFr rats

**FIGURE 3:**
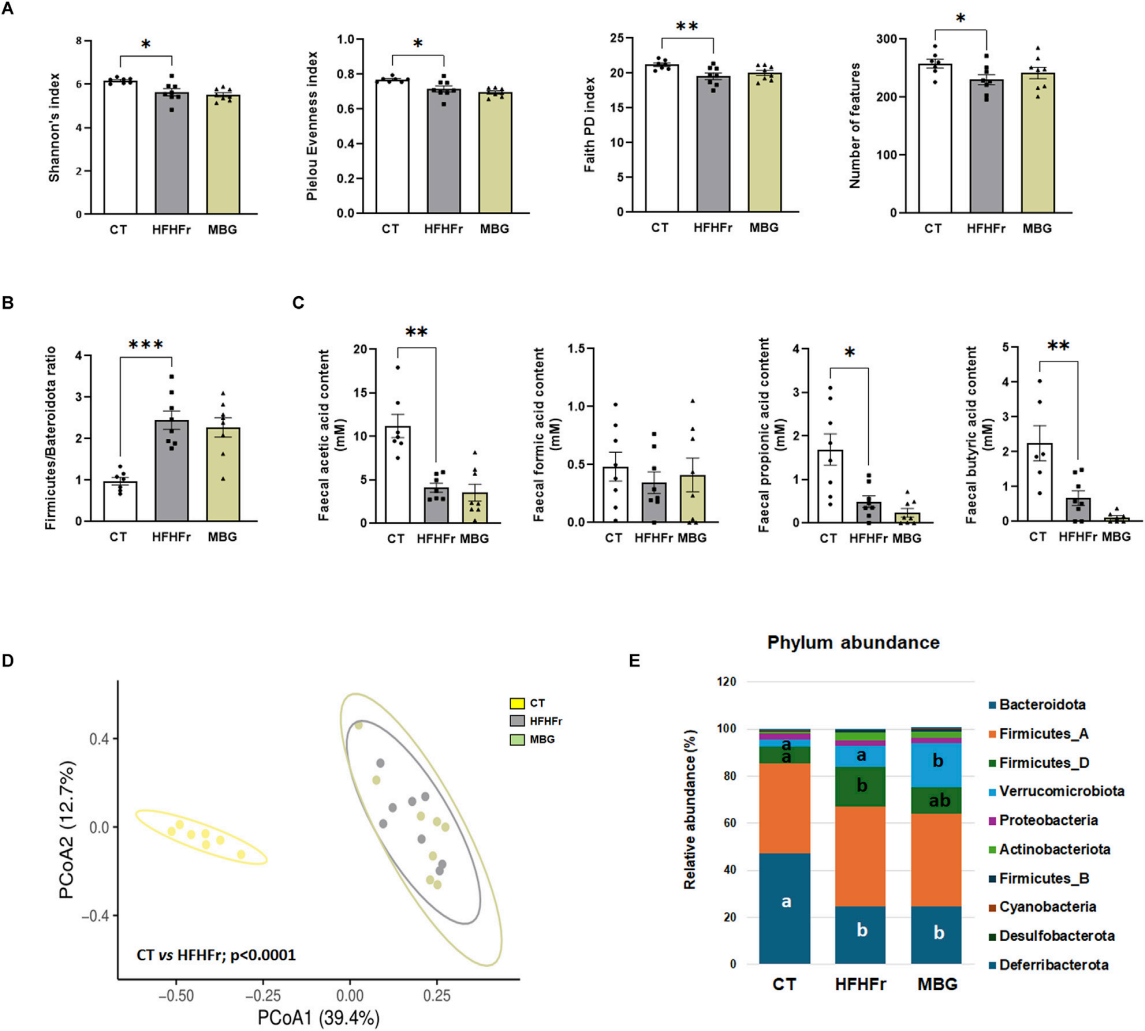
MBG administration did not change the biodiversity and evenness indexes, as well as the concentrations of SCFA in the faecal microbiota of HFHFr rats. **(A)** Alpha diversity indices: Shannon’s index, Pielou Evenness index, Faith Phylogenetic Diversity (PD) index, and number of features in stool samples from the CT, HFHFr and MBG experimental groups. **(B)** The ratio of Firmicutes to Bacteroidota in stool samples from the CT, HFHFr and MBG experimental groups. **(C)** Feacal SCFA concentrations in stool samples from the CT, HFHFr and MBG experimental groups. **(D)** Beta diversity analysis: Principal Coordinate Analysis (PCoA) based on Bray-Curtis dissimilarity. Significant p-values shown in the plot are derived from PERMANOVA (Permutational Multivariate Analysis of Variance) tests, highlighting significant differences between the groups, particularly between the control and HFHFr groups. **(E)** Phylum-level relative abundance of key bacterial groups in stool samples from the CT, HFHFr and MBG experimental groups. Data are presented as mean ±SEM, with individual values for each group (Control, HFHFr, and MBG). All data, except for PCoA, were analysed by ANOVA. In panel F, means with different letters are statistically different (P < 0.05). ​ *P < 0.05, **P < 0.01, ***P < 0.001.

**FIGURE 4:**
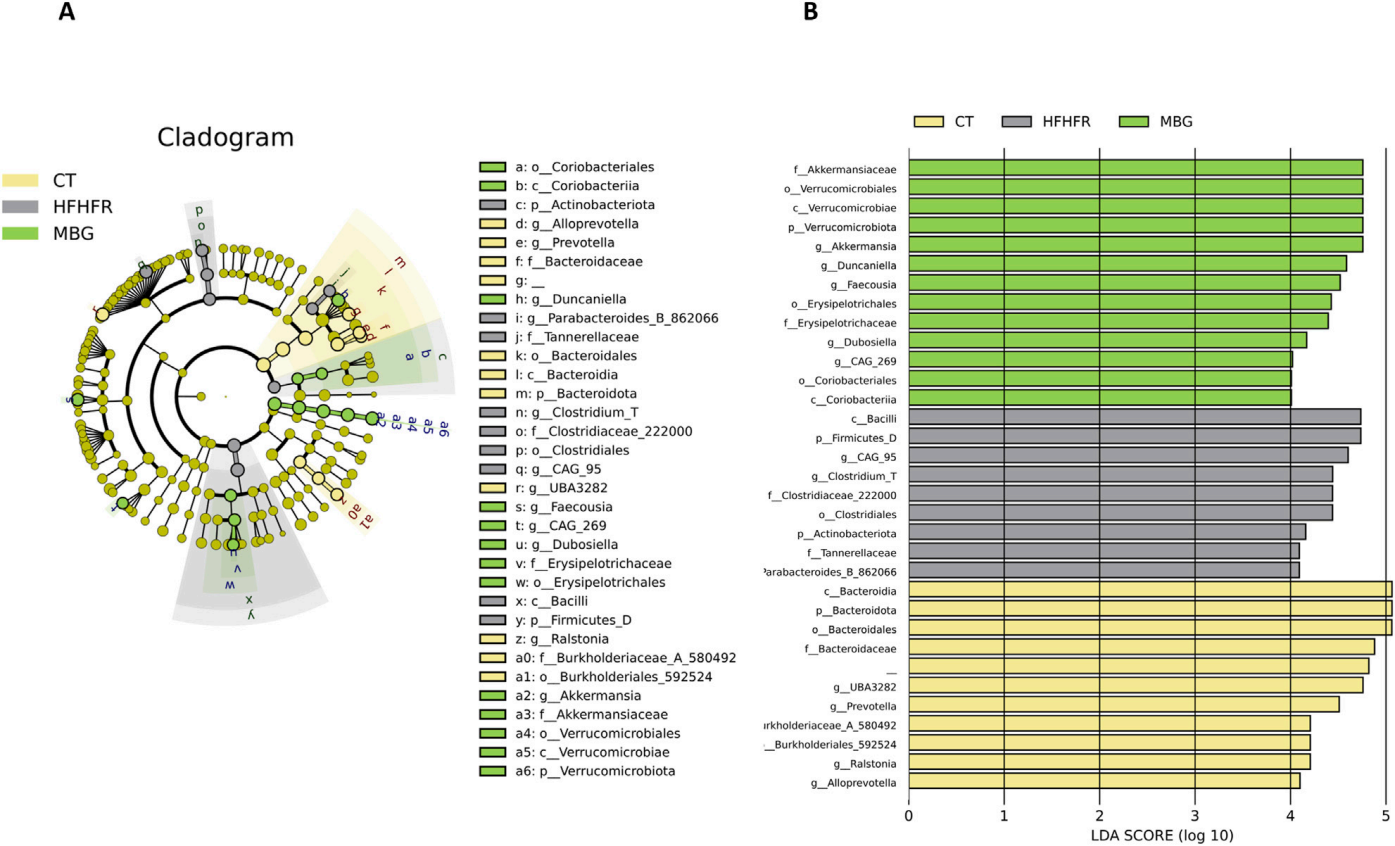
MBG modifies the relative composition of the faecal microbiota of female Sprague-Dawley rats fed with the HFHFr diet. **(A)** The cladogram and **(B)** the Logarithmic Discriminant Analysis (LDA) Effect Size (LEfSe) score plot demonstrate the differences in the relative abundance of bacterial taxa among the three groups. The phylogenetic tree illustrates the changes in the faecal microbiota down to the genus level induced by the HFHFr diet and MBG treatment. The coloured nodes represent significantly enriched taxa in each treatment group: yellow for Control, grey for HFHFr, and green for MBG. The bar chart on the right displays the LDA scores (log 10) for each taxon, indicating the effect size of their differential abundance among the groups. The taxa are listed on the y-axis, while the x-axis shows the LDA score. Higher LDA scores indicate a more significant difference in abundance. ​

**FIGURE 5:**
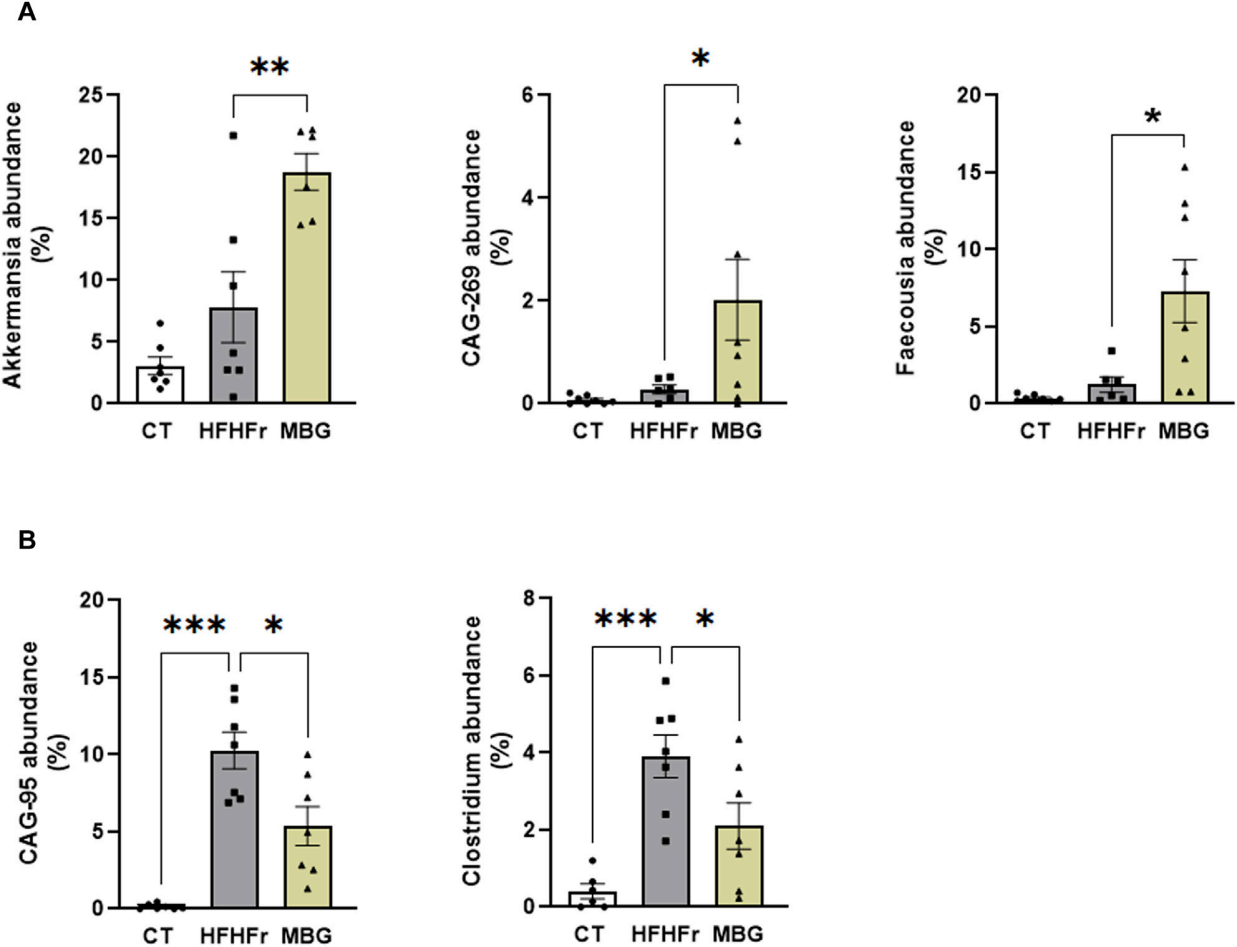
Select changes in the faecal microbiota composition of HFHFr rats after MBG treatment. **(A)** Relative abundance of *Akkermansia*, *CAG-269*, and *Faecousia* in faecal samples from CT, HFHFr, and MBG groups. **(B)** Relative abundance of *CAG-95* and *Clostridium* in faecal samples from CT, HFHFr, and MBG groups. Results are shown as bar plots with individual values, showing the mean ± SD of 7-8 animals/group. *P < 0.05, **P < 0.01, ***P < 0.001.

As previously reported, the consumption of the HFHFr diet reduced the biodiversity and evenness distribution of rat faecal microbiota, while increasing the relative abundance of Verrucomicrobiota, and Firmicutes, particularly Firmicutes D, and reducing the relative abundance of Bacteroidota. These changes were accompanied by a reduction in the faecal concentration of SCFA, with the exception of formic acid, which remained unchanged (Bentanachs et al., 2024). The qualitative parameters of the faecal microbiota, including biodiversity, evenness indexes and principal component analysis were unchanged by mirabegron administration to HFHFr rats (Figure 3). Similarly, the Firmicutes to Bacteroidota ratio, the relative phylum abundance and the faecal concentrations of SCFA remained unmodified (Figure 3). Nevertheless, Lefse analysis indicated that the drug treatment induced selective changes in the composition of the faecal microbiota (Figure 4). Specifically, MBG administration to HFHFr rats increased the relative abundance of the genera *Akkermansia* (Verrucomicrobiota), *CAG-269* and *Faecousia* (Firmicutes A), which were not significantly changed by the dietary intervention compared with controls (Figure 5A). On the contrary, the increased relative abundance of the genera *CAG-95* (Firmicutes A) and *Clostridium* (Firmicutes D), induced by the HFHFr diet consumption, was partially reverted by MBG administration (Figure 5B). These changes were not accompanied by significant changes in markers of colon epithelium integrity, such as the relative mRNA expression of the *occludin*, *tnfα* and *mucin2* genes (see Table 1).

**TABLE 1:** Relative mRNA expression in samples of rat colon mucosa from the different experimental groups. Data expressed as mean ± SD of 6-7 samples.

mRNA Arbitray units	CT	HFHFr	MBG
*Occludin*	100 ± 21	112 ± 34	97 ± 34
*Tnfα*	100 ± 31	58 ± 21	85 ± 41
*Mucin2*	100 ± 24	79 ± 19	78 ± 41

**FIGURE 6:**
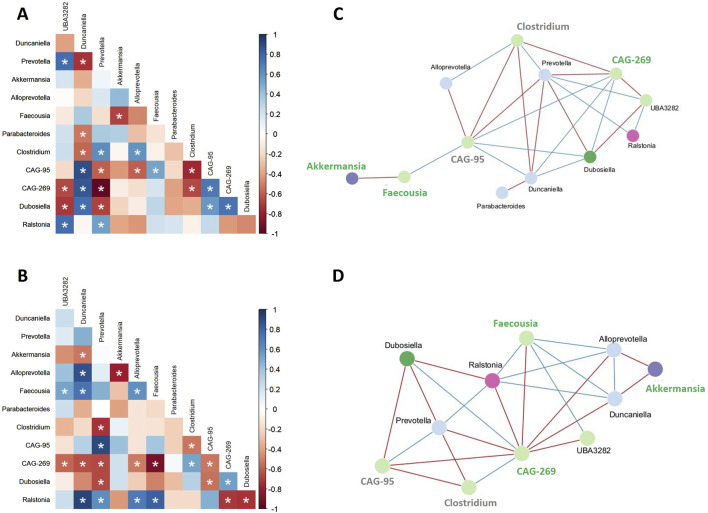
MBG administration modifies the microbiota co-occurrence in HFHFr rats. Correlation heatmaps **(A, B)** and microbial interaction networks **(C, D)** show the relationships among gut microbial genera in high-fat high-fructose (HFHFr) rats. Panels A and C represent untreated HFHFr animals, while panels B and D represent animals treated with MBG. Positive correlations are marked in blue, and negative correlations are marked in red, with statistically significant associations indicated by asterisks (P < 0.05). In panels C and D, blue lines represent positive interactions, and red lines represent negative interactions. Genera are color-coded according to their phylum classification. Those that increase after MBG treatment are highlighted in green, while those that decrease after treatment are marked in grey.

To assess associations between bacterial taxa within the faecal microbiota of each group, Spearman’s rank correlation was used to create co-occurrence networks, considering only significant correlations (rho >0.6 and p < 0.05, Figures 6A, B). A positive correlation indicates cooperative or interdependent relationships between taxa, while a negative correlation suggests a competitive relationship. Network analyses revealed structural differences between the HFHFr- and MBG-treated groups (Figures 6C, D). In HFHFr animals, the network was characterised by a few densely connected nodes, such as *Prevotella* and *CAG-95*, indicating dominant microbial cores. In contrast, MBG-treated animals showed a more balanced microbial network, with connections more evenly distributed among taxa, including *Faecousia* and *CAG-269*. To better quantify the structural differences observed in the microbial networks, the clustering coefficient and network density were calculated for both groups. The clustering coefficient, which quantifies the tendency of nodes to form tightly interconnected clusters, was higher in MBG-treated animals (0.769) than in HFHFr-treated animals (0.546). Similarly, network density, which indicates the ratio of actual connections to all possible connections, was higher in MBG-treated animals (0.455) than in HFHFr-treated animals (0.364). These results suggest that MBG treatment improves the connectivity and clustering of microbial communities.

## 4 Discussion

In the present work, by analysing biological samples from our previous study in a rat experimental dietary model of isolated steatosis (Bentanachs et al., 2024), we are able to demonstrate that oral administration of MBG to HFHFr rats significantly altered the expression of several genera and families in the faecal microbiota, although without restitution to the global biodiversity and evenness indexes seen in control rats.

In our previous study, we showed that MBG was ineffective in reducing liver triglyceride deposition. Here we show that this lack of effect cannot be attributed to a deficit in the activation of the β3-AR pathway in the liver, as MBG treatment effectively increases the protein levels of β3-AR in this tissue. Nevertheless, BRL37344, an experimental β3-AR agonist not in clinical use, was shown to reduce liver and serum triglycerides in a dietary MASLD model of male Sprague-Dawley rats (Wang et al., 2020). Given this, we expected MBG to have similar effects in our simple liver steatosis female Sprague-Dawley rat model. Besides the different dosages (0.1 and 10 mg/kg for BRL37344 and MBG, respectively), and possible differences in potency and efficacy as β3-AR agonists, sexual dimorphism could explain the divergent outcome with the two β3-AR agonists. We have previously shown that male Sprague-Dawley rats have a higher liver PPARα-related fatty acid β-oxidation activity than females (Bentanachs et al., 2023). BRL37344s molecular structure has a close similarity to several well-known PPARα activators such as bezafibrate or fenofibrate (Harada et al., 2005; Balendiran et al., 2012). Given that the main hepatic effect of BRL37344 was an increase in the PPARα-driven fatty acid β-oxidation, it is feasible that its anti-steatotic effect could be elicited by a direct activation of PPARα that is unrelated to its β3 receptor agonistic activity. Mirabegron does not have the same structural similarity with fibrates; therefore, coupled with the use of female Sprague-Dawley in our MASLD model, this could explain the lack of anti-steatotic effect.

Another experimental β3-AR agonist, CL316243, was studied in HFD-fed mice maintained at thermoneutrality, which showed increased liver triglyceride levels (Xiao et al., 2015). Similar to our results, the compound had no significant effect on liver histology, weight or triglyceride contents. However, CL316243 treatment increased BAT tissue *ucp1* mRNA and the corresponding protein levels in this model, causing a reduction in adiposity and body weight (Xiao et al., 2015). It has been previously shown that the increase in cyclic AMP produced by the activation of β3-AR present in BAT increases the expression of the UCP1 protein, uncoupling mitochondrial combustion of fatty acids in BAT from the production of ATP, resulting in the activation of non-shivering thermogenesis (Cannon and Nedergaard, 2004). Our results also showed that MBG increased the UCP1 protein content in BAT, suggesting a stimulating effect of MBG on thermogenesis. However, in our model this activation of BAT did not result in a reduction of body weight or adipose tissue weight, probably because HFHFr-fed rats were already lean (Bentanachs et al., 2024). In another study, *foz/foz* mice (a model of MASH) showed improvement in hepatic steatosis after moderate caloric restriction combined with MBG (Poekes et al., 2019). The improvement was attributed to the sum of reduced hepatic lipogenesis and increased energy substrate burning in the BAT. Our results, showing increased UCP1 protein expression in BAT without improved steatosis, suggest that MBG effects in our model may not be potent enough to reduce liver lipid contents without a significant reduction in caloric intake.

Regarding WAT, our results show that MBG effectively increases the amount of β3-AR protein in pWAT. Stimulation of β3-AR in adipose tissue activates protein kinase A, which in turn catalyses the phosphorylation of HSL to increase lipolysis. In our model, MBG treatment did not result in increased HSL phosphorylation nor in increased ATGL protein levels, suggesting a desensitization of the pathway in white adipocytes due to the continued effect of the β3-AR agonist. However, increased β3-AR protein in pWAT and marginal increases in plasma glycerol and NEFA levels may indicate either a compensatory mechanism or an initial, acute effect of enhanced lipolysis which is lost after 1 month of treatment.

In our previous work, the administration of pemafibrate to HFHFr rats significantly altered the faecal microbiota. This was probably, at least in part, due to changes in the liver expression of genes that encode enzymes involved in the synthesis of primary BA and translated to significant changes in faecal BA composition (Bentanachs et al., 2024). Here we show that MBG treatment, despite not changing the global biodiversity and evenness indexes of the faecal microbiota, induced significant changes in the expression of several bacterial genera and phyla. However, MBG did not exert any significant effect on faecal BA, indicating that the effect of MBG on the rat faecal microbiota was not mediated by BA. Baskin et al. have reported changes in bile acid metabolism associated with a β3-AR-driven increase in gallbladder volume. This led to reductions in plasma concentrations of glycochenodeoxycholate, glycocholic acid, glycodeoxycholate, and taurodeoxycholic acids in healthy men treated with MBG. Given that rats are devoid of a gallbladder (Higashiyama et al., 2018), the fact that we were unable to detect significant changes in faecal BA composition in our HFHFr rat experimental model could be related to the absence of a MBG-mediated effect on gallbladder BA metabolism. Thus, our results suggest a direct effect of MBG on the rat faecal microbiota that was not mediated by BA.

The impact of MBG on microbial community dynamics may stem from its influence on colonic motility. β3-adrenergic receptors are expressed in the colon, and agonists like Vibegron have been shown to reduce colonic motility and stool frequency, leading to improved symptoms in irritable bowel syndrome patients (Mozaffari et al., 2024). Reduced motility could result in a more stable colonic environment, favouring the proliferation of certain taxa, such as *Akkermansia* and *Faecousia*, which were identified as central nodes in the microbial networks of MBG-treated animals.

Furthermore, MBG treatment was associated with higher clustering coefficients and network density, suggesting a more interconnected and cooperative microbial community structure. These network changes likely reflect improved microbial stability and functional integration. A higher clustering coefficient indicates the formation of tightly interconnected microbial clusters, which are crucial for maintaining ecosystem stability and resilience (Coyte et al., 2015). Similarly, the increased network density highlights enhanced microbial interactions, which could facilitate nutrient exchange, SCFA production, and protection against opportunistic pathogens (Berry and Widder, 2014).

We could not find published data about the metabolic or physiological relevance of changes in the relative abundance of the genera *CAG-269*, *Faecousia* and *CAG-95*. Regarding the increase in the relative abundance of *Akkermansia* and the decrease in *Clostridium* induced by MBG, it has been reported that the opposite situation is associated with MASLD (Quesada-Vázquez et al., 2020; Vallianou et al., 2022; Korobeinikova et al., 2023), suggesting a beneficial, or at least a non-detrimental effect of MBG on the progression of MASLD.

In summary, our present work clearly demonstrates that oral treatment with MBG, although unable to reduce liver triglyceride accretion and hypertriglyceridemia in an HFHFr rat experimental model of simple hepatic steatosis, can significantly and directly alter the abundance of several genera and phyla of rat faecal microbiota, favouring a more interconnected and cooperative microbial community structure. This effect is not mediated by changes in bile acids composition induced by drug treatment.

## Data Availability

The raw data supporting the conclusions of this article will be made available by the authors, without undue reservation.
